# Machine learning of large‐scale spatial distributions of wild turkeys with high‐dimensional environmental data

**DOI:** 10.1002/ece3.5177

**Published:** 2019-04-24

**Authors:** Annie Farrell, Guiming Wang, Scott A. Rush, James A. Martin, Jerrold L. Belant, Adam B. Butler, Dave Godwin

**Affiliations:** ^1^ Department of Wildlife, Fisheries and Aquaculture Mississippi State University Mississippi State Mississippi; ^2^ Warnell School of Forestry and Natural Resources and Savannah River Ecology Laboratory University of Georgia Athens Georgia; ^3^ Camp Fire Program in Wildlife Conservation State University of New York College of Environmental Science and Forestry Syracuse New York; ^4^ The Mississippi Department of Wildlife, Fisheries, and Parks Jackson Mississippi; ^5^ Mississippi Forestry Association Jackson Mississippi

**Keywords:** habitat suitability, maximum entropy, multicollinearity, predictive ecological niche models, random forests, regularization, support vector machines, wildlife management

## Abstract

Species distribution modeling often involves high‐dimensional environmental data. Large amounts of data and multicollinearity among covariates impose challenges to statistical models in variable selection for reliable inferences of the effects of environmental factors on the spatial distribution of species. Few studies have evaluated and compared the performance of multiple machine learning (ML) models in handling multicollinearity. Here, we assessed the effectiveness of removal of correlated covariates and regularization to cope with multicollinearity in ML models for habitat suitability. Three machine learning algorithms maximum entropy (MaxEnt), random forests (RFs), and support vector machines (SVMs) were applied to the original data (OD) of 27 landscape variables, reduced data (RD) with 14 highly correlated covariates being removed, and 15 principal components (PC) of the OD accounting for 90% of the original variability. The performance of the three ML models was measured with the area under the curve and continuous Boyce index. We collected 663 nonduplicated presence locations of Eastern wild turkeys (*Meleagris gallopavo silvestris*) across the state of Mississippi, United States. Of the total locations, 453 locations separated by a distance of ≥2 km were used to train the three ML algorithms on the OD, RD, and PC data, respectively. The remaining 210 locations were used to validate the trained ML models to measure ML performance. Three ML models had excellent performance on the RD and PC data. MaxEnt and SVMs had good performance on the OD data, indicating the adequacy of regularization of the default setting for multicollinearity. Weak learning of RFs through bagging appeared to alleviate multicollinearity and resulted in excellent performance on the OD data. Regularization of ML algorithms may help exploratory studies of the effects of environmental factors on the spatial distribution and habitat suitability of wildlife.

## 1 INTRODUCTION

Studies of the spatiotemporal distribution of resources that support organisms are indispensable for understanding the dynamics of animal populations, including avian populations, across space and time (Fuller, 2012). Habitat suitability is the likelihood that a species uses or occupies a particular habitat (Kearney, 2006). Habitat suitability models predict the likelihood of animal occurrences at a spatial location using abiotic and biotic environmental variables, thus quantifying the environmental conditions that may lead to species occurrence (Hirzel & Le Lay, 2008). Animals select habitats based on their ecological and physiological needs and resource availability (Fretwell & Lucas, 1969; Rosenzweig, 1981). Consequently, habitat and its ecological conditions selected by animals may represent a subset of the species' fundamental ecological niche, which is defined as the environmental conditions allowing populations of a species to persist and grow (Basille, Calenge, Marboutin, Andersen, & Gaillard, 2008; Hirzel & Le Lay, 2008; Hutchinson, 1957). Therefore, habitat suitability index (HSI) may predict the abundance or carrying capacity of animal populations (Weber, Stevens, Diniz‐Filho, & Grelle, 2017).

Ecological niche modeling (ENM), including habitat suitability modeling, has become a fundamental tool for understanding the spatial distribution and conservation of biodiversity. Habitat suitability models (HSMs) relate species occurrences to landscape variables or resource availability in space (Hirzel & Le Lay, 2008). Machine learning (ML) methods such as maximum entropy (MaxEnt), random forest (RF), and support vector machine (SVM) algorithms have been used to map wildlife habitat suitability with impressive predictive accuracy (Carrasco, Mashiko, & Toquenaga, 2014; Kampichler, Wieland, Calmé, Weissenberger, & Arriaga‐Weiss, 2010; Milanesi, Holderegger, Caniglia, Fabbri, & Randi, 2015; Phillips, Anderson, & Schapire, 2006). Maximum entropy is a principle to find a probability distribution, at which an event (e.g., species occurrence) occurs with the greatest uncertainty (e.g., maximizing the Shannon entropy), while being subject to some constraints that the statistical moments (e.g., mean and variance) of the distribution match with the sample moments of observations. MaxEnt can be parameterized for presence‐only (PO) data in a way equivalent to the Poisson point process model, a spatial statistical model for count data. Despite the lack of intuition, MaxEnt has become a benchmarking ENM (Elith et al., 2011; Phillips, Anderson, Dudík, Schapire, & Blair, 2017; Renner & Warton, 2013).

**Figure 1:**
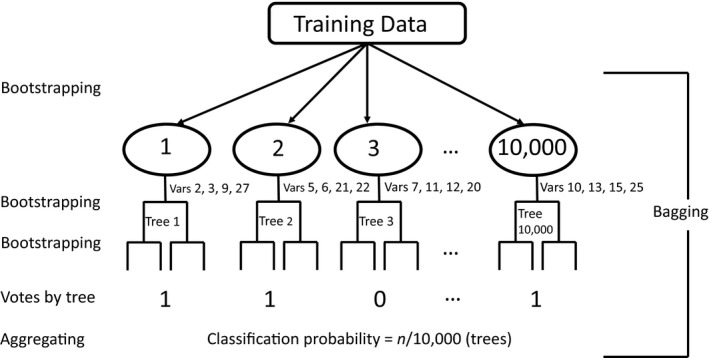
Illustration of the random forest algorithm. The bagging algorithm consists of bootstrapping and aggregating. Each oval represents a bootstrap sample from training data. The bootstrapping is implemented at each tree branching with a different random subset of covariates (Vars) until fit of each tree is optimized. Random forests aggregate “votes” over all trees to estimate classification probabilities

The RF algorithm draws a large number of random samples from the original data, fits classification and regression trees (CARTs) to each of the random samples, and then aggregates the “votes” or averages results over all the trees to make classifications or numeric predictions (Figure 1; Breiman, 2001). Random forests may achieve excellent performance for habitat suitability predictions unmatched by other ML methods through minimizing both the variance and bias of the models (Breiman, 2001; Kampichler et al., 2010). Support vector machines are a popular ML algorithm in pattern recognition due to the state‐of‐the‐art classification performance (Abe, 2005). Support vector machines deterministically choose support vectors (a subset of training data) as the boundary of a class in a high‐dimension feature space, and maximize separation between classes (See figure 8 of Wang, 2019 for a brief description and illustrations). Support vector machines also have been used to model animal habitat suitability (Drake, Randin, & Guisan, 2006; Fukuda & De Baets, 2016). Nonparametric inferences of RF, deterministic‐learning features of SVMs, and their excellent accuracy have made the two algorithms important, attractive tools for habitat suitability assessments (Drake et al., 2006; Evans, Murphy, Holden, & Cushman, 2011; Fukuda & De Baets, 2016).

Habitat suitability mapping often uses a large number of landscape variables (e.g., 10 or more variables) to predict habitat suitability. Many of those landscape variables are highly correlated to one another, leading to multicollinearity in habitat and resource selection models (Aebischer, Robertson, & Kenward, 1993; Cutler et al., 2007). Machine learning uses regularization, which shrinks the influences of redundant or overfitting predictors to zero, and bagging, which is bootstrapping aggregating (Figure 1), to overcome the curse of dimensionality. Random forests and SVMs are nonparametric, without relying on statistical distributions and specific parametric function forms, which endows ML advantages over generalized linear models, generalized additive models, and their variants for habitat modeling. Random forests use CART to account for nonlinear interactions between predictors and bagging to reduce dimensionality and alleviate multicollinearity (Breiman, 2001; Cutler et al., 2007). Support vector machines may not suffer from multicollinearity due to their deterministic solutions of support vectors (Drake et al., 2006). The program MAXENT implements the MaxEnt algorithm with an L‐1 regularization equivalent to the least absolute shrinkage and selection operator (LASSO) algorithm to avoid multicollinearity (Phillips et al., 2006). However, Merow, Smith, and Silander (2013) recommended to select a subset of noncorrelated covariates before using MAXENT. Assessments of the effectiveness and accuracy of MaxEnt, RFs, and SVMs for high‐dimensional data on large spatial scales can help guide ecologists to design ENMs.

There are two common statistical approaches to eliminating or reducing multicollinearity in HSMs (Merow et al., 2013). The first method is to remove one of two highly correlated variables (e.g., absolute Pearson correlation |*r*| > 0.7 or a higher cutoff value; hereafter correlation removal). The second method is to use the scores of orthogonal principal components, which explain the majority of variation in the original environmental variables (e.g., >90%; hereafter principal component approach). Drake et al. (2006) demonstrated that unprocessed data (their model 1) and orthogonal transformation (method 2) performed equally and better than correlation removal (method 3) in SVMs. Random forests may alleviate multicollinearity with a randomized subset of explanatory variables when growing each tree branch (Cutler et al., 2007). However, it is uncertain if MaxEnt differs in performance between using a subset of independent and all original environmental variables (Fukuda & De Baets, 2016; Merow et al., 2013). Few studies have compared the predictive accuracy among multiple ML methods such as MaxEnt, RFs, and SVMs with correlation removal and orthogonal transformation.

The Eastern Wild Turkey (*Meleagris gallopavo silvestris*; hereafter wild turkey) is the largest galliform in North America (Dickson, 1992). Wild turkeys select a variety of habitats, but are strongly associated with forests (Davis et al., 2017; Wang, 2018). Habitat selection by wild turkey in Mississippi has been well studied at the population and within‐home‐range levels (Chamberlain, Leopold, & Burger, 2000; McKinney, 2013; Miller & Conner, 2007; Miller, Leopold, Hurst, & Gerard, 2000). Wild turkeys exhibited an optimal response to increasing hardwood forests, with their relative abundance peaking at or leveling off (i.e., following a S‐shaped response curve beyond about 29% hardwood forest within landscapes) (Davis et al., 2017). The S‐shaped response curve of habitat use to increasing resource or habitat available is a form of nonlinear functional response of habitat or resource selection (Mysterud & Ims, 1998). To our knowledge, no study of wild turkey habitat assessment using either rigorous statistical models or ML methods on a regional scale (>100,000 km^2^), such as the entire state of Mississippi (*ca*. 125,443 km^2^), has been reported in the literature. In this study, we first developed statewide habitat suitability maps with a large sample size of presence data (e.g., 600–700 presence locations) using MaxEnt, RFs, and SVMs. Second, we compared predictive performances of MaxEnt, RFs, and SVMs between correlation removal and principal component approaches to multicollinearity. Ecological studies have not exploited extensively the excellent performances of SVMs in pattern identification and recognition and the capacity to analyze large amounts of data and complex relationships (Huettmann et al., 2018).

## 2 METHODS

### 2.1 Study area

Mississippi is located in the southeastern United States (US; 30.18341–34.99627 N, 91.63314–88.10944 W). Mississippi has a flat topology with elevation ranging from 0 to *ca*. 245 m a. s. l. Mean annual temperatures ranged from 16.67 to 18.33°C, and mean annual precipitation ranged from 127 to 165.1 cm. About 48% of land within Mississippi was covered by forests, including hardwood forests (i.e., deciduous trees as the dominant form of vegetation), pine forests, and pine‐hardwood mixed forests. The Mississippi Alluvial Valley region in westcentral Mississippi was dominated by agriculture, with only *ca*. 19% of land being covered by remnant bottomland hardwood forests (See Davis et al., 2017 for the description of vegetation).

### 2.2 Presence data

We acquired 763 presence locations of wild turkey from the following sources: (a) wild turkey trapping locations in January, February, and March of 2009 and 2010 (*n* = 17); (b) male bird harvest locations in March and April of 2014 (*n* = 74) and 2015 (*n* = 91); (c) brood surveys of females and young of the year birds in June, July, and August of 2014 (*n* = 288) and 2015 (*n* = 202); and (d) random sightings across the state throughout the year (*n* = 91). Cooperative turkey hunters recorded the geographic coordinates (longitude and latitude) of harvest locations on data sheets, which were designed and distributed by the senior author before the turkey hunting seasons (from mid‐March to 01 May), using a hand‐held global positioning system (GPS) unit. Impromptu sightings occurred when wildlife biologists of the Mississippi Department of Wildlife, Fisheries, and Parks (MDWFP) conducted routine work. Geographic coordinates of other sighting locations were determined using high‐resolution (15 m) Google Earth© Map (http://www.earth.google.com). Brood surveys were conducted by the MDWFP wildlife biologists in June, July, and August. Wild turkey broods with females were detected ~100–150 m from observers. Geographic coordinates of detected broods were recorded using a hand‐held GPS unit. Location errors (i.e., distance between detected broods and observers) were less than the 250‐m resolution of the land cover and land use (LCLU) maps used in our study. Additionally, frequency, edge density, and distance of land covers were generated as averages within a 1,785‐m circular buffer, which is the radius of average annual home range of wild turkeys in Mississippi (Davis et al., 2017). Thus, the effects of possible location errors (<200 m) were minimized by the spatial resolution of the landscape variables used in this study. We treated different sources of presence data equally because all types of data indicated the presence of wild turkeys in a certain life stage.

**Figure 2:**
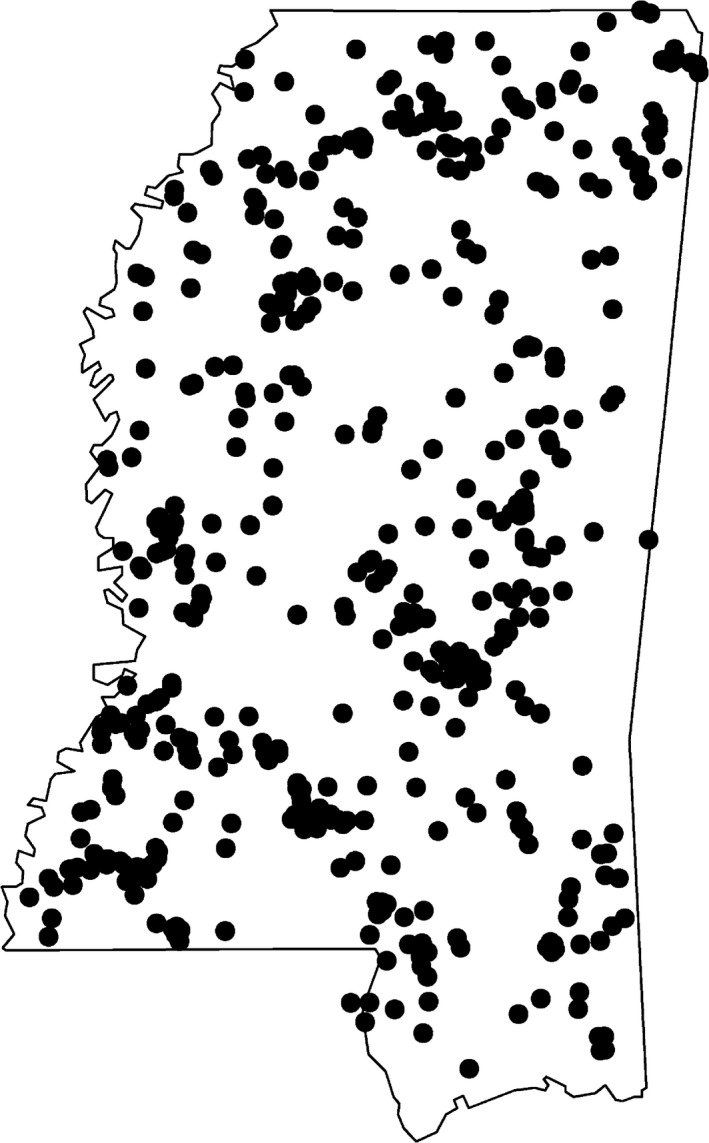
Spatial distribution of 453 presence locations of eastern wild turkey within the state of Mississippi, United States. The polygon is the boundary of Mississippi (in latitude and longitude). Black dots are nonduplicated location, with distance between any two locations being ≥2 km

A total of 663 nonduplicated locations were used for HSI mapping. To reduce spatial redundancy of presence locations, we randomly sampled presence locations with distances between any pairs of locations being >2 km using the R package *spThin* (Aiello‐Lammens, Boria, Radosavljevic, Vilela, & Anderson, 2015). The random selection by *spThin* resulted in 453 presence locations between any pairs of which distance was >2 km (Figure 2). Mean daily maximum movement distance of wild turkeys ranges from 1 to 2 km (Marable, Belant, Godwin, & Wang, 2012). Four hundred fifty‐three locations were used as training data for HSMs. The remaining 210 nonduplicated presence locations were used as test/validation data for MaxEnt, RFs, and SVMs to evaluate predictive performance.

### 2.3 Landscape data preparation

We created 27 landscape variables from the 2011 National Land Cover Database (NLCD) satellite imagery classified by the Multi‐Resolution Land Characteristics Consortium (http://www.mrlc.gov/). Mississippi LCLU types included 15 classes: open water, developed open space, developed low intensity, developed medium intensity, developed high intensity, barren land, hardwood forest, pine forest, mixed forest, shrub/scrub, grassland/herbaceous, pasture/hay, cultivated crops, woody wetland, and emergent herbaceous wetlands (Fry et al., 2011). The four classes of the type "developed" and "barren land" were combined to a single class, “developed.” We further combined hardwood forest with woody wetland into hardwood forest and grassland with pasture/hay as grassland to create nine LCLU classes.

We generated 250‐m LCLU raster maps (or layers) by resampling from the original 30‐m LCLUs to reduce computational burdens. We then derived three landscape variables for each of the nine LCLU classes: distance to the nearest grid cell (m), relative frequency (0–1.0), and edge density (m/ha), producing a total of 27 landscape variables (hereafter the original data [OD]). Distance layers were generated using the program Biomapper module DistAn (Hirzel, Hausser, Chessel, & Perrin, 2002). Frequency and edge density layers were generated in a radius of seven 250 m × 250 m grid cells using the Biomapper module CircAn (Hirzel et al., 2002). The radius of seven grid cells is equivalent to the average home range of wild turkeys in Mississippi (ca. 1,000 ha; Marable et al., 2012; Davis et al., 2017). Graf, Bollmann, Suter, and Bugmann (2005) found that landscape variables averaged over an annual home‐range buffer had the best predictive performance for capercaillie (*Tetrao urogallus*) habitat suitability modeling compared to other spatial scales.

We fit MaxEnt to the presence location data, and fit RFs and SVMs to the same presence locations and the same number of pseudo‐absence locations with the 27 original landscape variables, orthogonally transformed landscape data, and collinearity‐removed data separately to assess the impact of multicollinearity on the HSM performance. We used principal component analysis (PCA) to transform the original 27 landscape variables to principal components (hereafter PC data), which were orthogonal to one another, to avoid multicollinearity among original landscape variables. PC data were generated using the geographic information system (GIS) software IDRISI 15.0 (Clark Labs, Worcester, Massachusetts, USA), which generates the raster images of PCs in the same file format as programs CircAn and DistAn.

We used variance inflation factor (VIF) to remove landscape variables which were highly correlated with other landscape variables, decreasing the extend of multicollinearity (Neter, Kutner, Nachtsheim, & Wasserman, 1996). We used a VIF cutoff of 3.0 (>3.0) to exclude a variable (Graham, 2003; Zuur, Ieno, & Elphick, 2010). We used the R package *uSDM* to calculate VIFs of 27 landscape variables (Naimi, 2017; Naimi, Hamm, Groen, Skidmore, & Toxopeus, 2014), and termed the resulting subset of landscape variables reduced data (RD).

### 2.4 Habitat suitability models

MaxEnt models use a large number of randomly selected pseudo‐absence locations as background locations to quantify available resources (Elith et al., 2011; Merow et al., 2013). We used 10,000 randomly generated pseudo‐absence locations as recommended by Merow et al. (2013). We built MaxEnt models with the OD, PC, and RD data, respectively, using the R package *Dismo* with the default parameter settings of the program MaxEnt (Hijmans, Phillips, Leathwick, & Elith, 2017; Phillips et al., 2006).

Random forests and SVMs for 2‐class classification require absence locations for HSM. Ecological niche factor analysis (ENFA) uses environmental conditions including landscape variables at presence locations to quantify the multi‐dimensional ecological characteristics of the occupied habitat (Hirzel et al., 2002). Then, ENFA applies the multivariate profile or kernel to the entire landscape to generate a habitat suitability map without absence locations (Hirzel et al., 2002). As a multivariate statistical approach, the ENFA method also accounts for multicollinearity among landscape variables (Hirzel et al., 2002). Instead of randomly selecting pseudo‐absence locations, we first used ENFA to generate habitat suitability maps of wild turkeys only with 453 presence locations. Then, we randomly selected 453 pseudo‐absence locations restricted to the areas of low HSI away from the presence locations of wild turkeys with an approach similar to Senay, Worner, and Ikeda (2013).

We used Box‐Cox transformation to normalize 27 landscape variables for ENFA (Hirzel et al., 2002). We conducted ENFA for generating a statewide habitat suitability map of wild turkeys using the function *enfa* in the R package *adehabitatHS* (Calenge, 2006). To generate 453 pseudo‐absence locations for training RFs and SVMs, we followed the methods of Hengl, Sierdsema, Radović, and Dilo (2009) to calculate a composite weight of the ENFA‐predicted HSI and gridded buffer distance to observed occurrence locations using regression‐kriging. Pseudo‐absence locations were randomly selected at the composite weight of each 250 m × 250 m grid cell, and were located in the grid cell of low HSI away from observed presence locations (see Hengl et al., 2009 for the details). We generated 453 background locations for training and 210 background locations for evaluating RFs and SVMs.

We fit RFs to the three sets of landscape data (i.e., OD, PC, and RD), respectively, with 453 presence locations (coded as 1's) and 453 selected pseudo‐absence locations (coded as 0's) using the R package *randomForest* (Liaw & Wiener, 2002). We set the number of random trees (*n*) to 10,000. We used the default value of the parameter *mtry* (i.e., the number of randomly selected covariates). At last, RFs aggregate the results over 10,000 trees to make predictions, taking the majority of the votes of 10,000 trees for classification (Figure 1). We used RFs to classify a location to class presence or absence. We also used function *partialPlot* to plot the partial dependence of habitat occurrence probability on the logit scale on hardwood forest proportion, distance to hardwood forests, and hardwood forest edge density.

We used the Gaussian radial basis kernel for SVMs. We fit SVMs to the three sets of landscape data (i.e., OD, PC, and RD), using the function *svm* of the R package *e1701* (Meyer et al., 2018) and the same training data of 453 presence and 453 pseudo‐absence locations.

### 2.5 Accuracy assessment of HSI models

We evaluated the predictive accuracy of ENFA, RF, MaxEnt, and SVM predictions using the same test data (210 nonduplicated presence locations) with the continuous Boyce index (CBI; Boyce, Vernier, Nielsen, & Schmiegelow, 2002; Hirzel, Lay, Helfer, Randin, & Guisan, 2006). The CBI is a Spearman correlation between the predicted‐to‐expected (P/E) ratio of the habitat suitability value and mean HSI (Hirzel et al., 2006). The CBI ranges from −1 to 1, with 0 being equivalent to random predictions and a negative value indicating a wrong model (Hirzel et al., 2006).

We also used area under the curve (AUC) index from receiver operating curve (ROC) to assess the accuracy of ENFA, MaxEnt, RFs, and SVMs (Hilden, 1991; Liu, White, & Newell, 2011). The ROC is a curve of true positive rate (i.e., sensitivity) against false positive rate (i.e., 1‐specificity). The AUC ranges from 0 to 1, with 0.5 being equivalent to random predictions (Hilden, 1991). Accuracy is greater with a higher AUC (Liu et al., 2011). We used the function *evaluate* of the R package *Dismo* to calculate the AUC values for ENFA, MaxEnt, RFs, and SVMs.

We also determined the HSI threshold by maximizing the sum of the true positive rate and true false negative rates of each habitat suitability model using the function *evaluate*. We generated Boolean maps of suitable habitat, having the value 1 or 0 for a grid cell with its suitability index being greater or less than the threshold.

## 3 RESULTS

The first 15 principal components (PCs) explained 90% of variability in the original 27 landscape variables. The variable inflation factors (VIF) of 14 original landscape variables were greater than the cutoff of three and were excluded from the reduced data (RD, Appendix Table A1).

The AUC and CBI of the ENFA were 0.861 and 0.573, respectively, suggesting good fit. Maximum entropy, RFs, and SVMs with the PC all had excellent predictive accuracies (AUC and CBI >0.9) with RFs slightly over performing MaxEnt and SVMs (Table 1). Continuous Boyce indices indicated that all three classifiers performed equally well for the original data (OD) and RD data compared to the PC data (CBI >0.9). Nevertheless, AUC values demonstrated a slightly lower predictive performance of MaxEnt and SVMs for the OD data than the PC data, with the AUC value being 0.88 and 0.87, respectively, for the OD data (Table 1).

**Table 1:** The area under curve (AUC) and continuous Boyce index (CBI) of maximum entropy (MaxEnt), random forests (RF), and support vector machines (SVM) for the habitat suitability of wild turkeys in Mississippi, USA

Data set	MaxEnt‐CBI	RF‐CBI	SVM‐CBI	MaxEnt‐AUC	RF‐AUC	SVM‐AUC
PC	0.99	0.99	0.93	0.92	0.95	0.90
OD	0.97	0.91	0.97	0.88	0.92	0.87
RD	0.99	0.98	0.98	0.90	0.95	0.93

Notes

Symbol “OD” stands for the original data, “PC” for principal component, and “RD” for reduced data with correlated covariates being removed.

**Figure 3:**
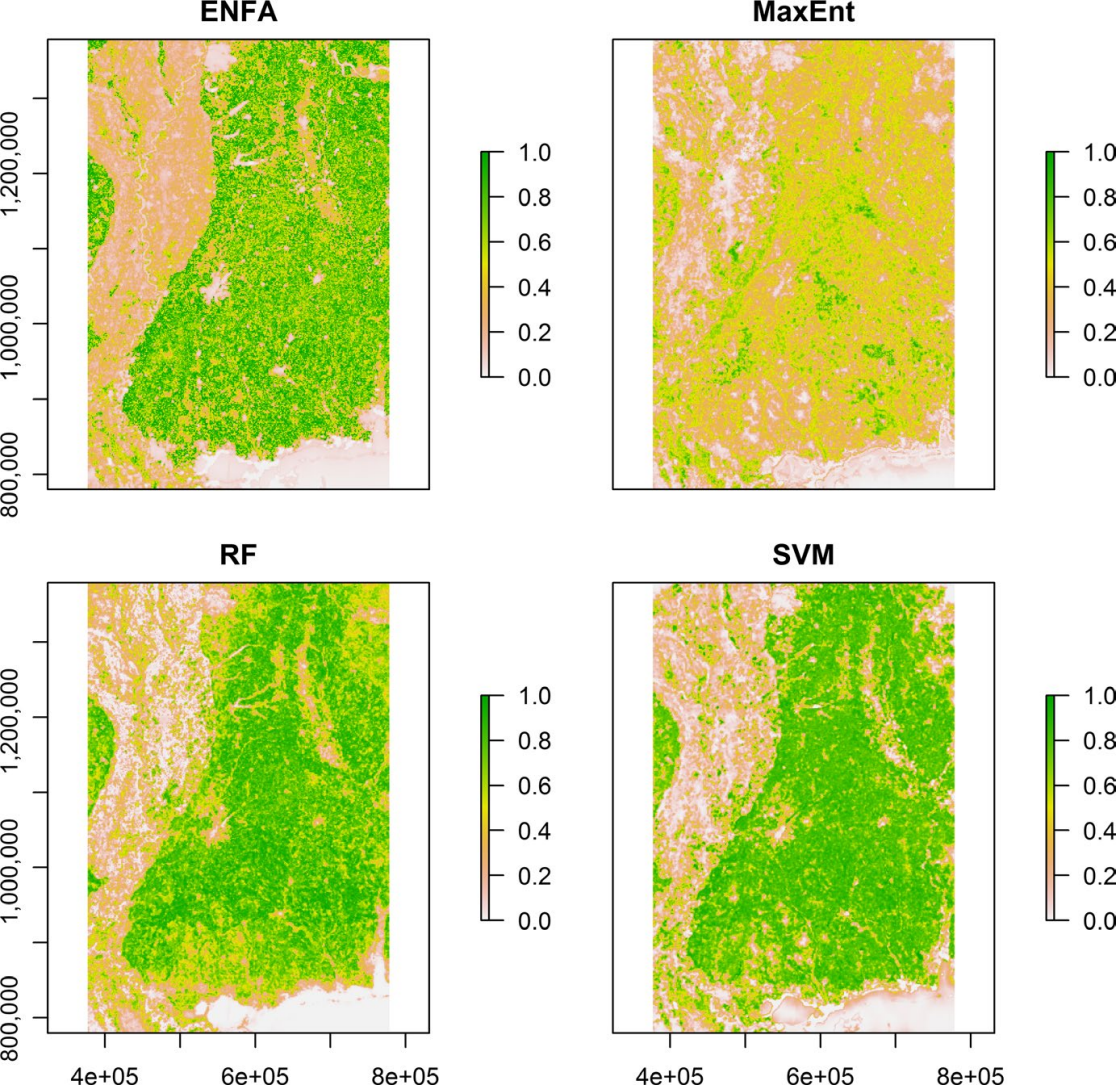
Habitat suitability maps of wild turkeys in Mississippi, USA, predicted by ecological niche factor analysis (ENFA, upper left panel), maximum entropy (MaxEnt, upper right panel), random forests (RF, lower left panel), and support vector machines (SVM, lower right panel)

**Figure 4:**
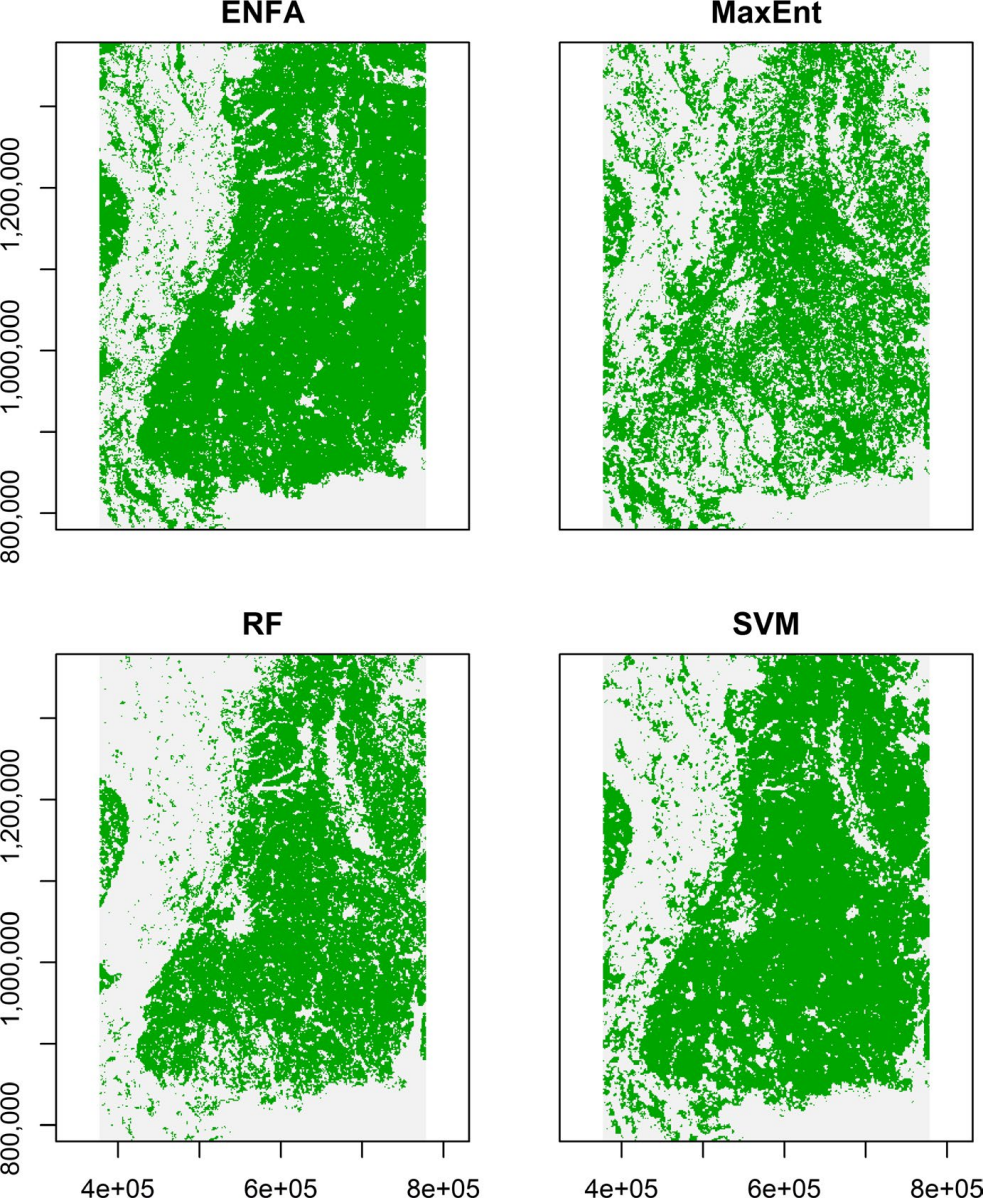
Boolean maps of suitable wild turkey habitats in Mississippi, USA, predicted by ecological niche factor analysis (ENFA, upper left panel), maximum entropy (MaxEnt, upper right panel), random forests (RF, lower left panel), and support vector machines (SVM, lower right panel). Green color represents suitable areas above a habitat suitability index (HSI) threshold

The three ML algorithms and ENFA predicted similar spatial distribution patterns of wild turkey habitats across Mississippi although the ranges of relative probabilities differed among methods (Figures 3, 4). Environmental niche factor analysis had excellent CBI values. Thus, pseudo‐absence locations generated by the regression‐kriging based on ENFA were primarily located in less suitable areas.

**Figure 5:**
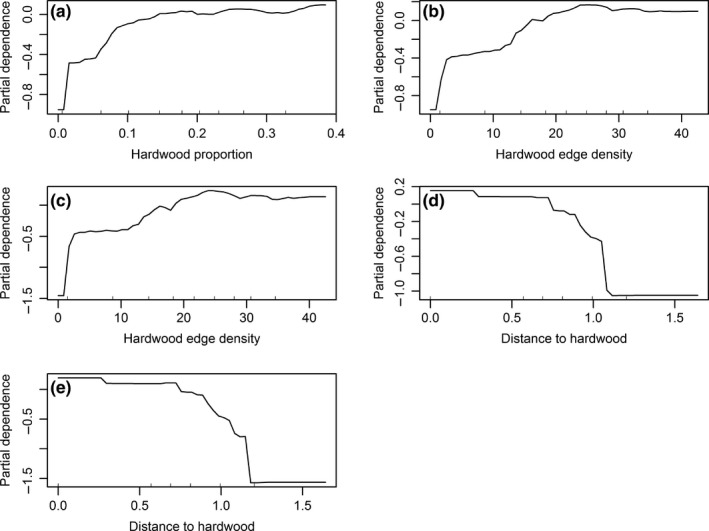
Partial plot of the partial dependence of the logit of occurrence probability of wild turkeys on (a) hardwood forest amount of full Random Forest models, (b) hardwood forest edge density of full Random Forest models, (c) hardwood forest edge density of simple random forest models, (d) distance to hardwood forests of full Random Forest models, and (e) distance to hardwood forests of simple random forest models in Mississippi, USA. The partial dependence was calculated with all other predictors being accounted for

The partial‐dependent effect of hardwood forest proportion on the occurrence probability of wild turkeys was nonlinear, increasing with increasing proportion and reaching an asymptote beyond 0.20 (Figure 5a). The RF models with the OD and RD data demonstrated the similar partial‐dependent effects of hardwood edge density (Figure 5b, c) and distance to hardwood forests (Figure 5d, e).

## 4 DISCUSSION

This study assessed the effectiveness of two different methods of correlation removal and principal component approaches to address multicollinearity on the predictive performance of Maximum entropy (MaxEnt), random forests (RFs), and support vector machines (SVMs) for habitat suitability modeling. Neither multicollinearity nor correlation removal reduced the predictive performance of MaxEnt, RFs, and SVMs substantially. Additionally, partial‐dependent effects of distance to hardwood forest and hardwood forest edge density are consistent between the RF models using the original data with multicollinearity and the reduced data of independent predictors. The occurrence of wild turkeys exhibited an increase and then level‐off with increasing hardwood proportion and edge density (i.e., functional response of habitat selection). Low amounts of hardwood forest and edge density appeared to limit the habitat use of wild turkeys. Nevertheless, the benefits of increasing hardwood forests and edge density leveled off or became saturated at high levels, consistent with the prediction of the functional response hypothesis for animal habitat selection (Mysterud & Ims, 1998).

Machine learning (ML) has various algorithms to combat the curse of dimensionality and multicollinearity including regularization and bagging. MaxEnt developed by Phillips et al. (2006) used the L‐1 regularization to account for multicollinearity in habitat/landscape variables. Our findings indicated that regularization with the MaxEnt default setting was sufficient to account for multicollinearity of the original data set of 27 landscape variables, of which 14 variables exhibited multicollinearity (Appendix Table A1). Despite the high predictive performance of MaxEnt models demonstrated in this study, to understand relationships between habitat selection by animals and landscape structure, the complexity and multicollinearity of MaxEnt models may need to be adjusted for robust, general inferences (Morales, Fernández, & Baca‐González, 2017). Francis et al. (2017) determined the optimal complexity of MaxEnt models for American beaver by selecting variables with Akaike's information criterion and relative contribution to model fit, tuning the *β* parameters for regularization, and removing correlative variables following Jueterbock, Smolina, Coyer, and Hoarau (2016). Francis et al. (2017) and this study have demonstrated the excellent predictive performance of HSMs using the PCs of landscape variables as predictors. However, the main disadvantage of using PC is the difficulty to interpret the effects of landscape structure on habitat selection, as a PC is a linear combination of original landscape variables.

Random forests may outperform SVMs and MaxEnt in ecological classification primarily because of the bagging algorithm (Breiman, 2001; Cutler et al., 2007), although no substantial performance differences were found among the three algorithms in this study. This study demonstrated excellent predictive performance of RFs with the original data of collinearity. Random forests may alleviate multicollinearity through bagging, which reduces the variance and bias of models simultaneously (Breiman, 2001; Cutler et al., 2007). Bagging has been increasingly used in ecological niche and species distribution modeling (Drake, 2014, 2015). Our findings suggested that the relationship between habitat selection and hardwood forest edge density was consistent between the simple and complex RF models (Figure 5), making RFs a useful tool for exploratory studies of the effects of environmental factors on spatial distributions of wildlife without facing difficulties of variable selection. Nevertheless, the collinearity of predictors may bias the outcome of variable selection (i.e., removing or retaining a variable) of RFs, diluting the relative importance of the variables of interest by redundant/overfitting variables (Murphy, Evans, & Storfer, 2010; Strobl, Boulesteix, Zeileis, & Hothorn, 2007). Furthermore, we here demonstrated the effectiveness of the three ML algorithms for multicollinearity of predictors for species distribution models (SDMs) with only one case study; thus, future studies may need to test and confirm the effectiveness of ML algorithms for multicollinearity in SDMs for different data and different ecosystems.

Support vector machines use the L‐2 regularization, minimizing the loss function of classification and regularizing term, which controls model complexity, based on statistical learning theory without requiring statistical distribution assumptions (Abe, 2005). Support vector machines generalize the inference/classification results only on the Vapnik–Chervonenkis (VC) dimension *h*, a reduced dimensionality of input data, to achieve sparsity. This study demonstrated robust predictive performance of SVMs to landscape data of collinearity like Drake et al. (2006). Additionally, the deterministic approaches may make SVMs faster and less costly in computation than RFs. Support vector machines are less popular than MaxEnt and RFs in the literature of species distribution models (Huettmann et al., 2018). Future studies may consider single‐class SVMs, a variant of SVMs for single‐class data, as a true presence‐only model for estimating species distributions (Mack & Waske, 2017).

Maximum entropy, RFs, and SVMs predicted the similar general patterns of wild turkey habitat distributions in Mississippi (Figures 3, 4). For instance, the region dominated by agriculture, grasslands such as the Black Prairie belt, and urban or developed areas had less suitable wild turkey habitats compared to the forested regions in Mississippi. However, boolean maps indicated that RFs and SVMs predicted more continuous habitats than MaxEnt models (Figure 4). The MaxEnt predictions captured isolated suitable habitats in the batture land east of the Mississippi River and along the river drainages (the upper right panels of Figures 3,4). Despite the similar patterns demonstrated by the three ML algorithms, the ranges of habitat suitability differed between MaxEnt and the other two methods probably because MaxEnt used much more randomly selected background locations than RFs and SVMs. Fukuda and De Baets (2016) demonstrated that data prevalence may affect the estimated range of habitat suitability and habitat suitability assessment. Ensemble approaches to integrating multiple HSMs into habitat suitability assessments may improve the robustness of HS predictions (Araújo & New, 2007).

Occurrence probabilities of wild turkey were also limited by low hardwood forest edge density below about 30 m edge/ha (Figure 5). Davis et al. (2017) found that the presence of diverse land covers, arranged in proximity to one another, enhanced relative abundance of wild turkeys, with increasing forest edges. Wild turkeys need agricultural fields, pastures, and forest openings for courtship and brood rearing (Hurst & Dickson, 1992; Speake, Lynch, Fleming, Wright, & Hamrick, 1975). Braunisch and Suchant (2007) found that small forest openings and small fields had positive effects on forest‐dwelling capercaillie (*Tetrao urogallus*). In our study, hardwood forest edge density served as a surrogate for the relative simultaneous access to both hardwood forests and different land covers that wild turkeys may have found within their home ranges. Landscapes of <20% or >30% hardwood forests may lack diversity, which reduced hardwood edge density, and thereby negatively affected the occurrence probability and potential abundance of wild turkey.

The abundance–suitability relationship may be positive in wildlife, including birds and mammals (Weber et al., 2017). The positive relationship may be ascribed to the same environmental variables favorable to both the occurrence and abundance of wildlife (Weber et al., 2017). Association of wild turkeys with forests has previously been recognized (Chamberlain et al., 2000; Davis et al., 2017). During the nesting season, females typically associate with managed pine (*Pinus* sp.) or hardwood forests (Miller & Conner, 2005; Miller, Hurst, & Leopold, 1999), whereas males prefer hardwood and pine forests (Miller et al., 1999). Davis et al. (2017) identified a parabolic relationship between relative male turkey abundance and proportion of hardwood forest, with relative abundance peaking in the habitat of 29% hardwood forest. This study used the presence data of male and female birds and found that the relative probability of occurrence of wild turkeys leveled off when the proportion of hardwood forest was more than 20%. The relationships illustrated from this study indicate that wild turkey populations in Mississippi may be limited by low amounts of hardwood forest at local scales. Nevertheless, abundance–suitability relationships may be complex (Dallas & Hastings, 2018). For instance, abundance may be low or high in the habitat of high suitability, with suitability predicting the upper limit of abundance or the carrying capacity of wild turkeys (Acevedo et al., 2017). Although we only presented the partial plots of RFs in this study, similar partial plots of SVMs and response curves or plots of MaxEnt can be used to examine the relationship between environmental variables and habitat suitability (Elith et al., 2011; Muñoz‐Mas, Fukuda, Pórtoles, & Martinez‐Capel, 2018; Phillips et al., 2006). Machine learning is a promising tool for species distribution modeling due to its nonparametric approaches and sparsity to overcome difficulties arising from high dimensions of environmental data and sparse data on occurrence, particularly in rare, threatened or endangered species.

## CONFLICT OF INTEREST

The authors have no conflict of interest related to this work.

## AUTHOR'S CONTRIBUTION

GW and AF conceived the ideas. AF, GW, and DG designed the study. AF collected data. DG and AB coordinated and participated in statewide data collection. AF and GW analyzed data. AF drafted the manuscript. All authors contributed to writing, revising, and improving the manuscript and gave the final approval for publication.

## Data Availability

Data on the presence of wild turkey used in this study are included in Supporting information.

## APPENDIX 1

**Table A1:** Variance inflation factor (VIF) of 27 landscape variables and the step VIF (VIFstep) of 14 landscape variables with the variable of the highest VIF being removed at each step

Variables	VIF	VIF_step_
Crop edge density	8.04	1.91
Distance to crop	6.71	NA
Crop frequency	23.88	NA
Developed edge density	4.34	NA
Distance to developed	2.29	2.1
Developed frequency	6.16	1.7
Wetland edge density	3.25	1.55
Distance to wetland	3.47	NA
Wetland frequency	1.91	1.52
Grassland edge density	19.79	NA
Distance to grassland	4.4	NA
Grassland frequency	14.18	1.42
Hardwood forest edge density	4.93	2.18
Distance to hardwood forest	2.51	1.87
Hardwood forest frequency	10.39	NA
Mixed forest edge density	13.73	NA
Distance to mixed forest	4.25	NA
Mixed forest frequency	8.82	1.85
Pine forest edge density	27.18	NA
Distance to pine forest	7.49	NA
Pine forest frequency	15.91	2.73
Shrubland edge density	31.38	NA
Distance to shrubland	4.75	NA
Shrubland frequency	16.58	2.42
Water edge density	3.25	1.43
Distance to water	3.99	NA
Water frequency	6.97	2.52

Note

Step VIF is calculated iteratively until all remaining variables have VIF of <3.0.
