# Supplementary material for: Machine learning of large‐scale spatial distributions of wild turkeys with high‐dimensional environmental data
Source: Ecol Evol. 2019 Apr 24;9(10):5938–49. doi: 10.1002/ece3.5177 (PMC6540709; doi:10.1002/ece3.5177)
Supplement: Supplementary file 4 [file ECE3-9-5938-s004.docx]

**SUPPLEMENTARY MATERIALS**

1. The data of 453 presence locations were used to train MaxEnt, Random Forests, and Support Vector Machines for the habitat suitability of wild turkeys in Missisippi, United States. The data were saved in the file “presencelocationsWildtTurkeyMS.xlxs.” Colunm heads “Longitude” and “Latitude” are the geographic coordinates (longitude and latitude in deciml degree) of presence locations.
2. The data of 453 pseudo-absence locations were used to train Random Forests and Support Vector Machines for the habitat suitability of wild turkeys in Missisippi, United States. The data were saved in the file “absencelocationsWildtTurkeyMS.xlxs.” Colunm heads “Longitude” and “Latitude” are the geographic coordinates (longitude and latitude in deciml degree) of the pseudo-presence locations.
3. The data of 210 presence locations were used to test or validate MaxEnt, Random Forests, and Support Vector Machines for the habitat suitability of wild turkeys in Missisippi, United States. The data were saved in the file “presencetestingdataWildtTurkeyMS.xlxs.” Colunm heads “Longitude” and “Latitude” are the geographic coordinates (longitude and latitude in deciml degree) of presence locations.
